# Protocol for the generation and expansion of human iPS cell-derived ureteric bud organoids

**DOI:** 10.1016/j.xpro.2022.101484

**Published:** 2022-06-17

**Authors:** Makoto Ryosaka, Shin-Ichi Mae, Kenji Osafune

**Affiliations:** 1 Center for iPS Cell Research and Application (CiRA), Kyoto University, 53 Kawahara-cho, Shogoin, Sakyo-ku, Kyoto 606-8507, Japan; 2 Rege Nephro Co., Ltd., Med-Pharm Collaboration Building, Kyoto University, 46-29 Yoshidashimoadachi-cho, Sakyo-ku, Kyoto 606-8501, Japan

**Keywords:** Stem Cells, Cell Differentiation, Organoids

## Abstract

The ureteric bud (UB) is a kidney precursor tissue that repeats branching morphogenesis and gives rise to the collecting ducts (CDs) and lower urinary tract. Here, we describe protocols to generate iUB organoids from human iPSCs; iUB organoids repeat branching morphogenesis. We describe how to expand iUB-organoid-derived tip colonies and how to induce CD progenitors from iUB organoids. These organoids can be used to study CD development and potentially as a model of kidney and urinary tract diseases.

For complete details on the use and execution of this protocol, please refer to Mae et al. (2020).

## Highlights

• Protocol for ureteric bud (UB) organoids with repeated branching potential
• An expansion culture approach for UB tip colonies using hydrogel
• Differentiation of UB organoids into 2D and 3D collecting duct progenitors

## Abstract (editor highlights)

Publisher’s note: Undertaking any experimental protocol requires adherence to local institutional guidelines for laboratory safety and ethics.

## Graphical abstract

**Figure:**
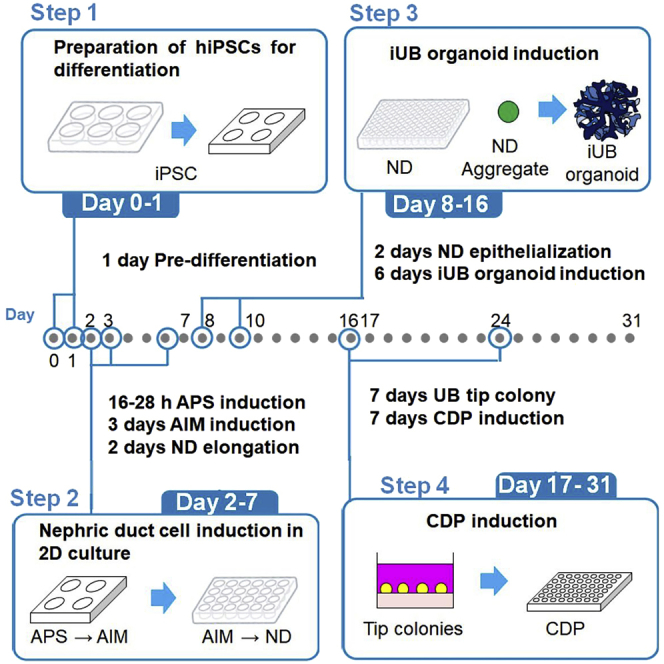

## Before you begin

***Note:*** All procedures in this protocol are performed in a Class II biological hood according to standard aseptic technique. Cells are cultured in a humidified 37°C incubator at 5% CO_2_.

### Institutional permissions

**Timing: up to 2 days**

Any animal experiments must be approved by the institutional animal care committee. Any experiments using human induced pluripotent stem cells (hiPSCs) must be approved by the institutional ethics committee and informed consent must be obtained from donors from whom hiPSCs were derived.

1. Preparation of stock solutions and aliquots.

Reconstitute the chemical compounds and growth factors according to the manufacturer’s recommendation and aliquot for storage (see materials and equipment for details).

## Key resources table

**Table:** 

REAGENT or RESOURCE	SOURCE	IDENTIFIER
**Antibodies**		

Mouse anti-AQP2 (1:200)	Santa Cruz	Cat#sc-515770; RRID: AB_2810957
Goat anti-BRACHYURY (1:500)	R&D	Cat#AF2085; RRID: AB_2200235
Mouse anti-CK8 (1:500)	Abcam	Cat#ab9023; RRID: AB_306948
Mouse anti-E-CADHERIN (1:500)	BD	Cat#610181; RRID: AB_397580
Goat anti-E-CADHERIN (1:200)	R&D	Cat#AF648; RRID: AB_355504
Mouse anti-EZRIN (1:500)	Abcam	Cat#Ab4069; RRID: AB_304261
Mouse anti-FOXA1 (1:500)	Santa Cruz	Cat#sc-514695
Rabbit anti-GATA3 (1:500)	Cell Signaling	Cat#5852S; RRID: AB_10835690
Goat anti-GATA3 (1:100)	R&D	Cat#AF2605; RRID: AB_2108571
Rabbit anti-LAMININ (1:500)	Sigma	Cat#L9393-2ML; RRID: AB_477163
Rabbit anti-PAX2 (1:500)	BioLegend	Cat#PRB-276P; RRID: AB_291611
Goat anti-PAX2 (1:500)	R&D	Cat#AF3364; RRID: AB_10889828
Rabbit anti-PRKCζ (1:200)	Santa Cruz	Cat#sc-216; RRID: AB_2300359
Goat anti-RET (1:500)	R&D	Cat#AF1485; RRID: AB_354820
Donkey anti-Mouse IgG- Alexa Fluor 488 (1:500)	Thermo Fisher Scientific	Cat#A21202; RRID: AB_141607
Donkey anti-Rabbit IgG- Alexa Fluor 488 (1:500)	Thermo Fisher Scientific	Cat#A21206; RRID: AB_2535792
Donkey anti-Goat IgG- Alexa Fluor 488 (1:500)	Thermo Fisher Scientific	Cat#A11055; RRID: AB_2534102
Donkey anti-Mouse IgG- Alexa Fluor 546 (1:500)	Thermo Fisher Scientific	Cat#A10036; RRID: AB_2534012
Donkey anti-Rabbit IgG- Alexa Fluor 546 (1:500)	Thermo Fisher Scientific	Cat#A10040; RRID: AB_2534016
Donkey anti-Goat IgG- Alexa Fluor 546 (1:500)	Thermo Fisher Scientific	Cat#A11056; RRID: AB_2534103
Donkey anti-Mouse IgG- Alexa Fluor 647 (1:500)	Thermo Fisher Scientific	Cat#A31571; RRID: AB_162542
Donkey anti-Rabbit IgG- Alexa Fluor 647 (1:500)	Thermo Fisher Scientific	Cat#A31573; RRID: AB_2536183
Donkey anti-Goat IgG- Alexa Fluor 647 (1:500)	Thermo Fisher Scientific	Cat#A21447; RRID: AB_2536183
Hoechst 33342 (1:500)	Thermo Fisher Scientific	Cat#H1399

**Chemicals, peptides, and recombinant proteins**		

A83-01	Wako	035-24113
30% w/v Albumin D-PBS(-) Solution, from Bovine Serum (BSA), Fatty Acid Free	Wako	015-23871
Accutase	Innovative Cell Technologies	AT104
Activin A	R&D	338-AC
Afamin/Wnt3a CM	MBL	J-ORMW301R
B-27 Supplement minus vitamin A	Thermo Fisher Scientific	12587001
Cell Recovery Solution	BD	354253
CHIR99021	StemRD	CHIR-010
DAPI	Merck	10236276001
DMEM/F-12, GlutaMAX	Thermo Fisher Scientific	10565042
D-PBS(-)	Nacalai Tesque	14249-24
EDTA	Thermo Fisher Scientific	15575020
EGF	R&D	236-EG-01M
Essential 6 Medium	Thermo Fisher Scientific	1516401
Fetal Bovine Serum	BIOSERA	FB-1285/500
FGF1	R&D	231-BC
FGF8	PeproTech	100-25
GDNF	R&D	212-GD
Geltrex	Thermo Fisher Scientific	A15696-01
iMatrix-511 silk	Nippi	892021
IWR-1	Merck	I0161-5MG
LDN193189	Axon Medchem	Axon1509
Matrigel	BD	354230
Normal donkey serum	Merck	566460
Penicillin-Streptomycin	Thermo Fisher Scientific	15140122
R-spondin 1	R&D	4645-RS-250
STEM-CELLBANKER GMP grade	ZENOAQ RESOURCE	ZR646
Stem Fit AK02N	Ajinomoto	AK02N
Sucrose	Nacalai Tesque	30403-55
Thiazovivin	Santa Cruz	SCB-SC-361380-10
Triton X-100	Nacalai Tesque	35501-15
TTNPB	Santa Cruz	sc-203303

**Experimental models: Cell lines**		

Human: 585A1 iPSC line	ICSCB	SKIP000858
Human: 1231A3 iPSC line	ICSCB	HPS0381
Human: 1383D2 iPSC line	ICSCB	HPS1005

**Software and algorithms**		

EZR	Kanda. 2013	http://www.jichi.ac.jp/saitama-sct/SaitamaHP.files/statmedEN.html
BZ-X Analyzer	KEYENCE	BZX700
R	The R Foundation	R version 3.6.1
ZEN 2 blue edition	ZEISS	ZEN 2.3 (blue edition)

**Other**		

Cell culture multiwell plate, 6 well	Greiner	657165
Cell culture multiwell plate, 24 well	Greiner	662160
Cell culture multiwell plate, 48 well	Greiner	677180
Multidish 4 well	Thermo Fisher Scientific	176740
Primesurface 35 mm	Sumitomo Bakelite	MS-90350
Primesurface 96 M	Sumitomo Bakelite	MS-9096M

## Materials and equipment

### Preparation and storage of growth factors and small-molecule compounds

#### 0.1% BSA in PBS to prepare stock solutions

Mix 100 μL 30% w/v Albumin D-PBS(-) Solution, from Bovine Serum (BSA), Fatty Acid Free (FUJIFILM) and 29.9 mL D-PBS(-). Collect the solution in a 50-mL syringe and filtrate it through a 0.22 μm filter. Store at 4°C for up to half a year.

#### Activin A

Centrifuge the vial prior to opening. Add 50 mL 0.1% BSA/PBS to 500 μg Activin A to prepare a 10 μg/mL stock. Mix by pipetting up and down. Make a 1 mL aliquot and store at -20°C for up to 1 year.

#### A83-01

Centrifuge the vial prior to opening. Add 474 μL DMSO to 2 mg A83-01 to prepare a 1 mM stock. Mix by pipetting up and down. Make a 100 μL aliquot and store at −20°C for up to 1 year.

***Note:*** You can also use A83-01 from EMD Bioscience (Cat# 616454-2MG).

#### CHIR99021

Centrifuge the vial prior to opening. Add 2.15 mL DMSO to 10 mg CHIR99021 to prepare a 10 mM stock. Mix by pipetting up and down. Make a 40 μL aliquot and store at −20°C for up to 1 year. To prepare 1 mM and 3 mM CHIR99021 working solution, add 360 μL and 94 μL DMSO, respectively. Store at 4°C for up to 3 months.

***Note:*** You can also use CHIR99021 from Wako (Cat# 038-23101).

#### EDTA

Add 500 μL UltraPure™ 0.5 M EDTA, pH 8.0, to 500 mL D-PBS(-) Filtrate the mixture through a 0.22 μm filter. Make a 50 mL aliquot and store at 4°C for up to 1 year.

#### EGF

Centrifuge the vial prior to opening. Add 10 mL 0.1% BSA/PBS to 1 mg EGF to prepare a 100 μg/mL stock. Mix by pipetting up and down. Make a 100 μL aliquot and store at −20°C for up to half a year.

#### FGF1

Centrifuge the vial prior to opening. Add 125 μL 0.1% BSA/PBS to 25 μg FGF1 to prepare a 200 μg/mL stock. Mix by pipetting up and down. Store at −20°C for up to half a year.

#### FGF8

Centrifuge the vial prior to opening. Add 5 mL sterile ddH_2_O to 1 mg FGF8 to prepare a 200 μg/mL stock. Mix by pipetting up and down. Make a 100 μL aliquot and store at −20°C for up to half a year.

#### GDNF

Centrifuge the vial prior to opening. Add 100 μL 0.1% BSA/PBS to 10 μg GDNF to prepare a 100 μg/mL stock. Mix by pipetting up and down. Store at −20°C for up to 1 year.

#### IWR-1

Centrifuge the vial prior to opening. Add 1.22 mL DMSO to 5 mg IWR-1 to prepare a 10 mM stock. Mix by pipetting up and down. Make a 100 μL aliquot and store at −20°C for up to 1 year. To prepare a 1 mM working solution, add 90 μL DMSO to the 10 mL stock solution. Store the working solution at 4°C for up to half a year.

#### LDN193189

Centrifuge the vial prior to opening. Add 388 μL DMSO to 2 mg LDN193189 to prepare a 10 mM stock. Mix by pipetting up and down. Make a 50 μL aliquot and store at −20°C for up to 1 year. To prepare a 0.1 mM LDN193189 working solution, add 99 μL DMSO to 1 μL of the 10 mM LDN193189 stock solution. Store the working solution at 4°C for up to half a year.

#### Matrigel

Thaw the Matrigel vial on a 4°C incubator for 24 h. Use precooled tips and precooled reaction tubes to make 2 mg aliquots. Store the aliquots at −20°C. The aliquots are stable for around 1 year (see certificate of analysis for each batch). Before use, thaw the required number of aliquots on ice (takes around 30 min).

***Note:*** Keep Matrigel always on ice and take it out of the ice only when it is to be added to the medium.

#### Thiazovivin

Centrifuge the vial prior to opening. Add 3.21 mL DMSO to 10 mg Thiazovivin to prepare a 10 mM stock. Mix by pipetting up and down. Make a 50 μL aliquot and store at −20°C for up to 1 year.

#### TTNPB

Centrifuge the vial prior to opening. Add 2.87 mL DMSO to 10 mg TTNPB to prepare a 10 mM stock. Mix by pipetting up and down. Make a 50 μL aliquot and store at −80°C until the expiry date indicated on the label. To prepare 0.1 mM TTNPB working solution, add 99 μL DMSO to 1 μL 10 mM TTNPB stock solution. Store the working solution at 4°C for up to half a year.

#### Y-27632

Centrifuge the vial prior to opening. Add 7.39 mL sterile ddH_2_O to 25 mg Y-27632 to prepare a 10 mM stock. Mix by pipetting up and down. Make a 400 μL aliquot and store at −20°C for up to 1 year.

#### R-spondin 1

Centrifuge the vial prior to opening. Add 1 mL 0.1% BSA/PBS to 100 μg R-spondin 1 to prepare a 100 μg/mL stock. Mix by pipetting up and down. Make a 100 μL aliquot and store at −20°C for up to half a year.

#### Afamin/Wnt3a CM

Thaw Afamin/Wnt3a CM at 4°C for 24 h. Make a 1 mL aliquot and store at −20°C for up to 1 year.

**CRITICAL:** Avoid repeated freeze-thaw cycles.

### Culture media

**Table:** Anterior primitive streak induction medium (APS medium)

Reagent	Final concentration	Amount
Activin A (10 μg/mL)	100 ng/mL	10 μL
CHIR99021 (3 mM)	3 μM	1 μL
Essential 6 medium	n/a	1 mL
Total	n/a	1 mL

n/a: not applicable.

Make APS medium fresh for every use.

***Note:*** 1 mL APS medium is required for two wells of a 4-well culture plate. We use two wells for a regular differentiation experiment.

**Table:** Anterior intermediate mesoderm induction medium (AIM medium)

Reagent	Final concentration	Amount
A83-01 (1 mM)	1 μM	1 μL
TTNPB (100 μM)	100 nM	1 μL
LDN193189 (0.1 mM)	0.1 μM	1 μL
FGF8 (200 μg/mL)	200 ng/mL	1 μL
Essential 6 medium	n/a	1 mL
Total	n/a	1 mL

n/a: not applicable.

Make AIM medium fresh for every use.

***Note:*** 1 mL AIM medium is required for two wells of a 4- or 24-well culture plate. We use two wells for a regular differentiation experiment.

**Table:** Nephric duct induction medium (ND medium)

Reagent	Final concentration	Amount
CHIR99021 (1 mM)	1 μM	2 μL
TTNPB (100 μM)	100 nM	2 μL
LDN193189 (0.1 mM)	0.1 μM	2 μL
FGF8 (200 μg/mL)	200 ng/mL	2 μL
GDNF (100 μg/mL)	100 ng/mL	2 μL
Essential 6 medium	n/a	2 mL
Total	n/a	2 mL

n/a: not applicable.

Make ND medium fresh for every use.

***Note:*** 2 mL ND medium is required for two wells of a 24-well culture plate. We use two wells for a regular differentiation experiment.

**Table:** UB organoid medium

Reagent	Final concentration	Amount
CHIR99021 (1 mM)	1 μM	2 μL
TTNPB (100 μM)	100 nM	2 μL
LDN193189 (1 mM)	1 μM	2 μL
FGF8 (200 μg/mL)	200 ng/mL	2 μL
GDNF (100 μg/mL)	100 ng/mL	2 μL
FGF1 (200 μg/mL)	200 ng/mL	2 μL
EGF (100 μg/mL)	50 ng/mL	1 μL
Essential 6 medium	n/a	2 mL
Matrigel	2% vol	40 μL
Total	n/a	2 mL

n/a: not applicable.

Make UB organoid medium fresh for every use.

***Note:*** 2 mL UB organoid medium is required for 20 wells of a 96-well culture plate. We use two wells for a regular differentiation experiment.

**CRITICAL:** Keep Matrigel on ice during the entire process. Before making the UB organoid medium, thaw the required number of Matrigel aliquots on ice for 30 min. Take them out of the ice only when they are to be added to the medium. After adding Matrigel to the medium, you can manipulate the UB organoid medium at around 25°C because the final Matrigel concentration is sufficiently low.

**Table:** Reconstituted UB organoid medium

Reagent	Final concentration	Amount
TTNPB (100 μM)	100 nM	2 μL
LDN193189 (1 mM)	1 μM	2 μL
FGF8 (200 μg/mL)	200 ng/mL	2 μL
GDNF (100 μg/mL)	100 ng/mL	2 μL
FGF1 (200 μg/mL)	200 ng/mL	2 μL
EGF (100 μg/mL)	50 ng/mL	1 μL
Essential 6 medium	n/a	1.8 mL
Matrigel	2% vol	40 μL
R-spondin1	200 ng/mL	2 μL
Afamin/Wnt3a CM	10% vol	200 μL
Total	n/a	2 mL

n/a: not applicable.

Make the reconstituted UB organoid medium fresh for every use.

***Note:*** 2 mL reconstituted UB organoid medium is required for 20 wells of a 96-well culture plate. We regularly use two wells for a differentiation experiment.

**CRITICAL:** Keep Matrigel on ice during the entire process. Before making the reconstituted UB organoid medium, thaw the required number of Matrigel aliquots on ice for 30 min. Take them out of the ice only when they are to be added to the medium. After adding Matrigel to the medium, you can manipulate the reconstituted UB organoid medium at around 25°C because the final Matrigel concentration is sufficiently low.

**Table:** UB tip medium

Reagent	Final concentration	Amount
CHIR99021 (3 mM)	3 μM	1.5 μL
TTNPB (100 μM)	100 nM	1.5 μL
Thiazovivin (10 mM)	10 μM	1.5 μL
FGF1 (200 μg/mL)	200 ng/mL	1.5 μL
GDNF (100 μg/mL)	100 ng/mL	1.5 μL
B27 supplement	2% vol	30 μL
DMEM/F12, GlutaMAX	n/a	1.5 mL
Total	n/a	1.5 mL

n/a: not applicable.

Make the UB tip medium fresh for every use.

***Note:*** 1.5 mL UB tip medium is required for 5 wells of a 48-well culture plate. We use 5 wells for a regular differentiation experiment.

**Table:** Collecting duct progenitor (CDP) induction medium (CDP medium)

Reagent	Final concentration	Amount
IWR1 (1 mM)	1 μM	2 μL
A83-01 (1 mM)	1 μM	2 μL
Essential 6 medium	n/a	2 mL
Total	n/a	2 mL

n/a: not applicable.

Make the CDP medium fresh for every use.

***Note:*** 2 mL CDP medium is required for two wells of a 24-well culture plate. We use two wells for a regular differentiation experiment.

**Table:** DMEM/F12 10%FBS

Reagent	Final concentration	Amount
DMEM/F-12, GlutaMAX	n/a	450 mL
Fetal Bovine Serum (FBS)	10 vol%	50 mL
Total	n/a	500 mL

n/a: not applicable.

**CRITICAL:** Store DMEM/F12 10%FBS at 4°C for up to 3 months.

***Note:*** After mixing DMEM/F12, GlutaMAX and FBS, filtrate the mixture through a 0.22 μm filter.

**Table:** Hydrogel

Reagent	Final concentration	Amount
DMEM/F-12, GlutaMAX	n/a	75 μL
Matrigel	50 vol%	75 μL

n/a: not applicable.

Make hydrogel fresh for every use.

**CRITICAL:** Keep Matrigel and hydrogel on ice during the entire process. Before making hydrogel, thaw the required number of Matrigel aliquots on ice for 30 min. Take them out of the ice only when they are to be added to the medium. After mixing the medium and Matrigel, you should keep hydrogel on ice to prevent it from solidifying until adding it on the culture plate.

***Note:*** Hydrogel is an appropriate culture environment in terms of stiffness for forming UB tip colonies.

***Note:*** Hydrogel with sufficient hardness cannot be formed with Geltrex.

## Step-by-step method details

### Maintenance of hiPSCs

**Timing: every 4 days**

This section describes the maintenance culture of hiPSCs.

1. Maintenance culture.
  a. Maintain the hiPSCs in feeder-free cultures containing Stem Fit AK02N medium on 6-well plates.
  b. Passage the hiPSCs every four days with no more than 80% confluence.
  c. To passage the hiPSCs, aspirate the medium and wash the cells with 2 mL EDTA (0.5 mM)/PBS. Add 2 mL EDTA/PBS and incubate the cells at 37°C for 3 min.
    **CRITICAL:** Extended exposure (> 10 min) to EDTA/PBS will decrease cell viability and differentiation potential when you passage hiPSCs.
  d. Aspirate the EDTA/PBS and add 2 mL Stem Fit AK02N. Dissociate the cells into single cells by gentle pipetting.
  e. After cell counting, replate the cells at a density of 5 × 10^4^ cells/well in 6-well plates and add 2 mL Stem Fit AK02N medium containing 10 μΜ Y-27632 and 0.125 μg/cm^2^ iMatrix-511 silk.
    ***Note:*** We recommend that maintenance medium for hiPSCs be Stem Fit AK02N or AK03N (Ajinomoto) because the induction efficiency of UB lineage cells may be decrease with hiPSCs maintained in other media, such as Essential 8 (Thermo Fisher Scientific).
    ***Note:*** We add iMatrix-511 silk to the medium to coat the plate at passaging. You may precoat the plate with iMatrix-511 silk at 37°C for 1 h; however, this is a time-consuming process (Miyazaki et al., 2017).
    **CRITICAL:** Adding ROCK inhibitor is important to prevent hiPSC apoptosis at passaging.

### Preparation of hiPSCs for differentiation

**Timing: 1 day for pre-differentiation**

This section describes the preparation culture of hiPSCs before differentiation.

**Figure 1:**
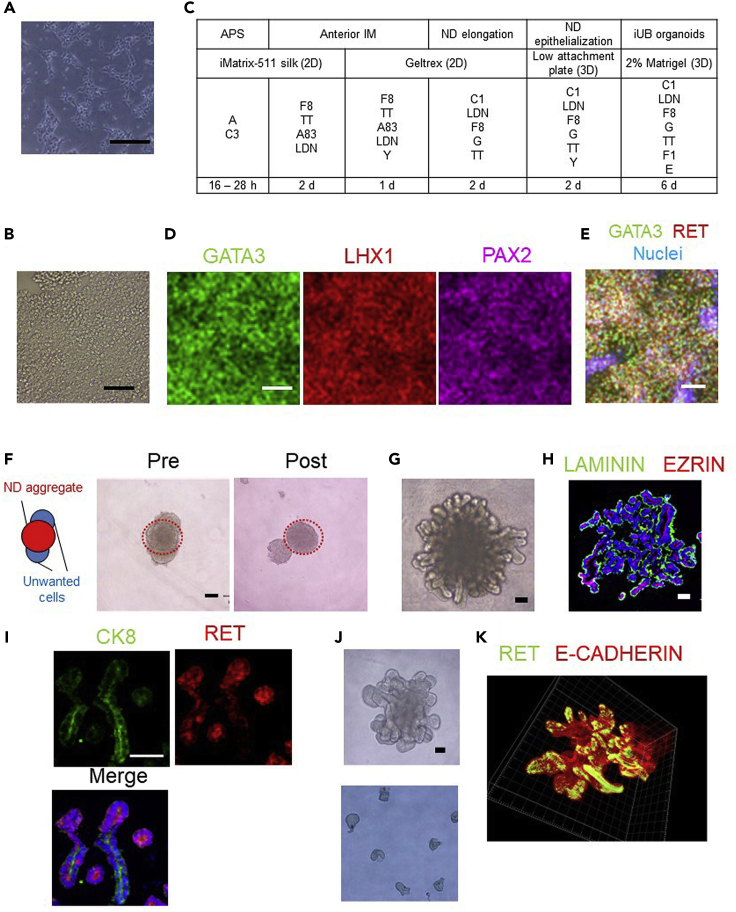
Induction of iUB organoids (A) Morphology of hiPSCs 24 h after thawing prior to differentiation. (B) Morphology of hiPSCs treated with EDTA/PBS for 3 min. The cells must have clearly defined bright edges under a brightfield microscope. (C) The protocol to differentiate hiPSCs into iUB organoids. APS, anterior primitive streak; AIM, anterior intermediate mesoderm; ND, nephric duct; UB, ureteric bud; A, 100 ng/mL Activin A; C3, 3 μM CHIR99021; F8, 200 ng/mL FGF8; TT, 0.1 μM TTNPB; A83, 1 μM A83-01; LDN, 0.1 μM LDN193189; Y, 10 μM Y-27632; C1, 1 μM CHIR99021; G, 100 ng/mL GDNF; F1, 200 ng/mL FGF1; and E, 50 ng/mL EGF. (D) Immunostaining of day 3 AIM cells for GATA3 (green), LHX1 (red) and PAX2 (purple). (E) Immunostaining of day 2 ND elongation cells for GATA3 (green), RET (red) and nuclei (blue). (F) A schematic showing a ND aggregate (red) and unwanted cells (blue; left panel) and morphology of a day 2 aggregate before (middle panel) and after (right panel) pipetting to remove unwanted cells. The red circles in the middle and right panels indicate a ND aggregate. (G) Morphology of a day 6 iUB organoid. (H) Immunostaining of a day 7 iUB organoid for LAMININ (green), EZRIN (red) and nuclei (blue). (I) Immunostaining of cryosections of a day 7 iUB organoid for CK8 (green), RET (red) and nuclei (blue). (J) Morphology of a day 6 iUB organoid (upper panel) and tips separated from day 6 iUB organoids (lower panel). (K) 3D whole-mount immunostaining of a day 14 separated tip-derived iUB organoid for RET (green) and E-CADHERIN (red). Scale bars, 300 μm in (F) and 100 μm in (A)–(E) and (G)–(J). (J) is adapted from Mae et al. (2020).

2. Pre-differentiation.
  a. Prepare the hiPSC suspension at passaging.
  b. After cell counting, replate the cells at a density of 2.5 × 10^4^ cells/cm^2^ and add Stem Fit AK02N medium containing 10 μΜ Y-27632 and 0.125 μg/cm^2^ iMatrix-511 silk.
    ***Note:*** hiPSCs will be 30%–40% confluency during pre-differentiation.
    **CRITICAL:** Both confluency and the size of the initial hiPSC colonies should be considered to avoid spontaneous differentiation.
    ***Note:*** We use two wells of a 4-well culture plate, and the medium volume is 500 μL for a regular differentiation experiment. Other plate formats, such as 24-well plates, can be used alternatively.
    **CRITICAL:** Adding ROCK inhibitor to the medium is important to prevent apoptosis at pre-differentiation.
  c. Incubate the cells at 37°C for 24 h (Figure 1A).
    ***Note:*** Incubate the cells with EDTA/PBS until the cells have clearly defined bright edges under a brightfield microscope (Figure 1B). The differentiation efficiency might decrease if the cells are treated with EDTA/PBS for >5 min or too short.
    ***Note:*** We recommend that the maintenance medium for hiPSCs be Stem Fit AK02N or AK03N (Ajinomoto) because the induction efficiency of UB lineage cells may decrease with hiPSCs maintained in other media, such as Essential 8 (Thermo Fisher Scientific).

### Nephric duct cell induction in 2D culture

**Timing: 16–28 h for APS induction, 3 days for AIM induction and 2 days for ND elongation**

This section describes the induction of nephric duct (ND) cells through APS and AIM steps.

3. APS induction (Figure 1C).
  a. Prepare 1 mL APS medium for two wells of a 4-well plate before starting this step.
  b. After 1 day of pre-differentiation with StemFit AK02N, wash the cells with 500 μL PBS and add 500 μL APS medium per well.
  c. Incubate the cells at 37°C for 16–28 h.

***Note:*** You may need to optimize the incubation time depending on the hiPSC lines. To confirm APS induction, we recommend bright field microscopic observations and immunostaining analysis. Refer to troubleshooting 1.

4. AIM induction (Figure 1C).
  a. Prepare 3 mL AIM medium before starting this step.
  b. After 16–28 h of APS induction, wash the cells with 500 μL PBS, add 1 mL AIM medium per well and incubate at 37°C for 2 days without changing the medium.
  c. After 2 days, coat the 24-well plates with 300 μL Geltrex per well and incubate at 37°C for 1 h.
    ***Note:*** Geltrex is ready-to-use and stored at 4°C until the expiry date indicated on the label. Coat new 24-well plates with undiluted Geltrex. You can use Matrigel alternatively.
  d. Aspirate the AIM medium and wash the cells with 500 μL PBS.
  e. Add 300 μL Accutase per well and incubate the cells at 37°C for 3 min.
    **CRITICAL:** Extended exposure (>3 min) to Accutase will lead to decreased cell viability.
  f. Detach the cells from the plates and dissociate them into single cells by manual pipetting about ten times.
  g. Collect all cell suspensions from all wells in a 15-mL tube and add DMEM/10% FBS up to 1.5 mL/well in the 4-well plates to stop the Accutase activity.
  h. Centrifuge the cells at 200×*g* for 5 min and aspirate the medium.
  i. After cell counting, replate the cells at a density of 1.0 × 10^5^ cells/cm^2^ in a Geltrex-coated 24-well plate with 500 μL AIM medium containing 10 μΜ Y-27632 and incubate at 37°C for an additional 24 h.
    ***Note:*** Immunostaining analysis for GATA3, LHX1 and PAX2 can be performed to confirm AIM induction 72 h later. (Figure 1D). AIM cells co-express these three markers.

5. ND elongation (Figure 1C).
  a. Prepare 2 mL ND medium to correspond to two wells of a 24-well plate before starting this step.
  b. Wash the AIM cells with 500 μL PBS and add 1 mL ND medium per well of the 24-well plate.
  c. Incubate the cells at 37°C for 2 days without a medium change.

***Note:*** Immunostaining analysis for RET can be performed to confirm ND elongation cell induction. ND elongation cells co-express RET and GATA3 (Figure 1E).

### iUB organoid induction in 3D culture

**Timing: 2 days for ND epithelialization and 6 days for iUB organoid induction**

**Timing: 6 days for the reconstitution of iUB organoids**

This section describes the induction of iUB organoids through ND epithelialization step and reconstitution of iUB organoids from separated tip regions of iUB organoids.

6. ND epithelialization (Figure 1C).
  a. Prepare 2 mL ND medium for 20 wells of a 96-well plate before starting this step.
  b. Wash the ND cells with 500 μL PBS per well and then aspirate the PBS.
  c. Add 300 μL Accutase per well and incubate at 37°C for 3 min.
  d. Detach the cells from the plates and dissociate them into single cells by manual pipetting about ten times.
  e. Collect all cell suspensions in a 15-mL tube and add DMEM/10% FBS up to 1.5 mL/well in the 24-well plates to stop the Accutase activity.
  f. Centrifuge the cells at 200×*g* for 5 min and aspirate the medium.
    ***Note:*** You can cryopreserve these cells at a density of up to 2 × 10^6^ cells/mL with STEMCELL BANKER.
  g. After resuspending the cells in ND medium and cell counting, seed the cells at a density of 1 × 10^4^ cells/well in low-attachment M-bottom 96-well plates with 100 μL ND medium plus 10 μM Y-27632 and incubate the cells at 37°C for an additional 2 days without a medium change.
    ***Note:*** After two days, one ND aggregate including unwanted cells is formed per one well of 96-well culture plates.

7. iUB organoid induction (Figure 1C).
  a. Prepare 2 mL UB organoid medium for 20 wells of a 96-well plate before starting this step.
  b. Separate unwanted cells from ND aggregates by manual pipetting 10–20 times using a 10- or 200-μL pipette (Mae et al., 2018) (Figure 1F).
    ***Note:*** Unwanted cells are not UB lineage and arise in the process of UB differentiation because the induction rate of ND cells is not 100%. We recommend separating these unwanted cells to purify ND cells. The ND cell region is larger and harder than unwanted regions in one aggregate per well (Figure 1F).
    ***Note:*** If unwanted cells cannot be separated from the ND aggregate, reinduce the ND cells from hiPSCs by improving the APS induction step. Refer to troubleshooting 2.
  c. Aspirate ND medium and add 100 μL organoid medium per well to the same 96-well plates.
  d. Incubate the aggregates at 37°C for 6 days. Organoid medium is changed every two days to generate iUB organoids (Figures 1G–1I).
    ***Note:*** iUB organoids will be floating in the well. One iUB organoid is formed per well of the 96-well plates.
    **CRITICAL:** Excessive pipetting (over 30 times) will break ND aggregates and may decrease UB budding.
    ***Note:*** M-bottom 96-well plates with spindle-shaped bottoms are recommended to generate iUB organoids. It is difficult to generate ND cell aggregates with U-bottom 96-well plates and to observe cell aggregates by microscopy with V-bottom 96-well plates.
    ***Note:*** You may centrifuge M-bottom 96-well plates containing ND cells at 200×g for 2 min to promote the cell aggregate formation.
    ***Note:*** The induction rate of iUB organoids may be low if the budding regions of iUB organoids are reduced and/or thickened. Refer to troubleshooting 3.
    Additional: Reconstitution of iUB organoids from separated tip regions of iUB organoids.
  e. Prepare 2 mL UB organoid medium before starting this step.
  f. Move some iUB organoids to a 35-mm dish (Sumitomo Bakelite) with 2 mL Essential 6 medium.
  g. Prepare two 22G needles (Terumo, # NN-2225R) and a stereomicroscope.
  h. Manually separate the UB tip regions from iUB organoids with two needles using a stereomicroscope (Figure 1J).
  i. Move one separated UB tip region into one well of the low-attachment M-bottom 96-well plates and add 100 μL UB organoid medium per well.
  j. Incubate the aggregate at 37°C for 14 days. Organoid medium is changed every two days to generate iUB organoids (Figure 1K).
    ***Note:*** This reconstitution process can be repeated 3–4 times for each tip.

### 2D-CDP induction

**Timing: 7 days for UB tip colony induction and 7 days for CDP induction**

This section describes the induction of CDPs in 2D culture from iUB organoids through UB tip colonies.

**Figure 2:**
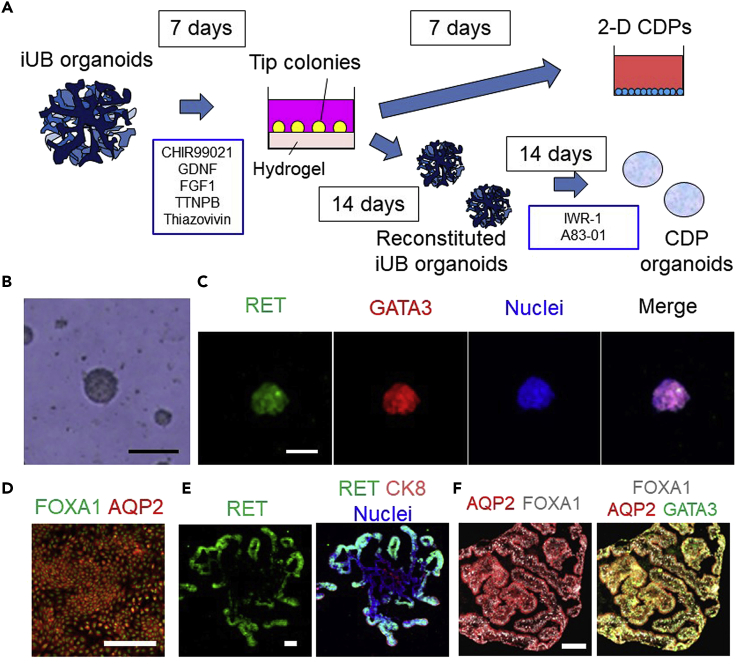
Induction of tip colonies and CDPs (A) A schematic showing the induction methods for tip colonies and CDPs. (B) Morphology of day 7 tip colonies. (C) Immunostaining of a day 7 tip colony for RET (green), GATA3 (red) and nuclei (blue). (D) Immunostaining of CDPs for FOXA1 (green) and AQP2 (red). (E) Immunostaining of a day 14 reconstituted iUB organoid for RET (green), CK8 (red) and nuclei (blue). (F) Immunostaining of a CDP organoid for GATA3 (green), AQP2 (red) and FOXF1 (white). Scale bars, 100 μm. (D) and (F) are adapted from Mae et al. (2020).

8. UB tip colony induction (Figure 2A).
  a. Prepare 600 μL UB tip medium for two wells of a 48-well plate before starting this step.
  b. Coat the 48-well plate with 150 μL/well hydrogel.
    ***Note:*** Keep Matrigel and hydrogel on ice to prevent them from solidifying before use. For details, refer to materials and equipment.
  c. Incubate the plate to solidify the hydrogel at 37°C for 1 h.
  d. Collect 10 day 6 iUB organoids in a 1.5 mL tube.
  e. Wash the organoids with 500 μL PBS twice.
  f. Aspirate PBS and add 100 μL Accutase. Incubate at 37°C for 5 min.
  g. Add 900 μL DMEM/F12 containing 10% FBS and dissociate the organoids by manual pipetting about 30 times.
  h. Centrifuge the cells at 200×*g* for 5 min and aspirate the medium.
  i. After resuspending the cells in 100 μL UB tip medium and cell counting, seed the cells at a density of 5 × 10^4^ cells/cm^2^ in 48-well plates and add UB tip medium up to 300 μL per well.
  j. Incubate the cells at 37°C for 7 days. Change the medium every two days.
    ***Note:*** After 7 days, 500–1,000 tip colonies 80–100 μm in diameter will be formed per well.
    ***Note:*** Immunostaining analysis for RET and GATA3 will help confirm the tip colony induction (Figures 2B and 2C). Tip colonies co-express these markers.
    ***Note:*** If UB tip colonies cannot be formed, refer to troubleshooting 4.

9. 2D-CDP induction (Figure 2A).
  a. Prepare 1 mL CDP medium for two wells of a 24-well plate before starting this step.
  b. Aspirate the medium of day 7 UB tip colonies in 48-well plates (see **8. UB tip colony induction of** step-by-step method details) and add 300 μL Cell Recovery Solution per well.
  c. Incubate the plate at 4°C for 1 h to dissolve hydrogel containing 50% Matrigel.
  d. Collect all cell suspension from the four wells of the 48-well plates into a 1.5 mL tube, centrifuge the colonies at 500×*g* for 5 min and aspirate the supernatant.
    ***Note:*** If the hydrogel remains around the UB tip colonies, we recommend adding 1 mL Cell Recovery Solution to the 1.5 mL tube after aspirating the supernatant. Pipet gently three to four times and incubate the cells at 4°C for an additional 1 h.
  e. Wash the cell pellet in the 1.5 mL tube with 1 mL PBS and centrifuge the colonies at 500×g for 5 min.
  f. Aspirate PBS and add 100 μL Accutase in the 1.5 mL tube containing the UB tip colonies. Incubate the cells at 37°C for 5 min.
  g. Add 900 μL DMEM/F12 containing 10% FBS per well and dissociate the colonies by manual pipetting until obtaining a single cell suspension.
  h. Centrifuge the cells at 200×*g* for 5 min and aspirate the medium.
  i. After resuspending the cells in 100 μL CDP medium and cell counting, seed the cells at a density of 5 × 10^4^ cells/cm^2^ in 24-well plates with 500 μL CDP medium containing 10 μΜ Y-27632 and 0.125 μg/cm^2^ iMatrix-511 silk.
    ***Note:*** We add iMatrix-511 silk to the medium to coat the plate. You may precoat the plate with iMatrix-511 silk at 37°C for 1 h; however, this is a time-consuming process (Miyazaki et al., 2017.).
  j. Incubate the cells at 37°C for 7 days. Change CDP medium every two days.
    ***Note:*** Immunostaining analysis for FOXA1 and AQP2 will help confirm CDP induction (Figure 2D). CDPs co-express these markers.
    ***Note:*** If the induction rate of CDPs is low, refer to troubleshooting 5.

### 3D-CDP organoid induction

**Timing: 14 days for reconstitution of iUB organoids and 14 days for 3D-CDP induction**

This section describes the induction of CDPs in 3D culture from UB tip colonies through reconstituted iUB organoids.

10. Reconstitution of iUB organoids from tip colonies (Figure 2A).
  a. Prepare 2 mL iUB organoid medium to correspond to 10 reconstituted iUB organoids before starting this step.
  b. Aspirate the medium of day 7 UB tip colonies (after finishing **8. UB tip colony induction of** step-by-step method details) and add 300 μL Cell Recovery Solution per well.
  c. Incubate the cells at 4°C for 1 h to dissolve the hydrogel.
  d. Collect all colony suspensions in a 15-mL tube, centrifuge the cells at 500×*g* for 5 min and aspirate the supernatant.
    ***Note:*** If the hydrogel remains around the UB tip colonies, we recommend adding 5 mL Cell Recovery Solution and incubating the cells at 4°C for an additional 1 h.
  e. Wash the colonies with 5 mL PBS twice.
  f. After resuspending the colonies in 2 mL iUB organoid medium, seed the colonies from 10 wells of 48-well plates to one low-attachment 35 mm dish.
  g. Incubate the colonies at 37°C for 14 days. Change iUB organoid medium every two days.
    ***Note:*** Immunostaining analysis for RET and CK8 may help confirm iUB organoid reconstitution (Figure 2E). The tip and trunk regions of reconstituted iUB organoids express RET and CK8, respectively.

11. 3D-CDP organoid induction (Figure 2A).
  a. Prepare 1 mL CDP medium for 10 3D-CDP organoids before starting this step.
  b. Aspirate the medium of day 14 reconstituted iUB organoids (after finishing **10. Reconstitution of iUB organoids from tip colonies of** step-by-step method details) and wash the organoids with 2 mL PBS.
  c. Aspirate PBS and add 2 mL CDP medium.
  d. Incubate the cells at 37°C for 14 days. Change CDP medium every two days.

***Note:*** Immunostaining analysis for FOXA1 and AQP2 will help confirm 3D CDP organoids induction (Figure 2F). 3D CDP organoids co-express these markers.

## Expected outcomes

The most important outcomes of these protocols include the induction of iUB organoids that have the potential for repeated branching morphogenesis, UB tip colony expansion and CDP differentiation. Immunostaining analysis is a useful method to evaluate the quality of the iUB organoids, UB tip colonies and CDPs. High quality iUB organoids express not only a basal marker, LAMININ, but also an apical marker, EZRIN, indicating that they have apicobasal polarity and tubular lumens (Figure 1H). Additionally, immunostaining for RET and CK8 is important for confirming the formation of the UB tip and trunk domains, respectively (Figure 1I). Immunostaining for RET and GATA3 and for FOXA1 and AQP2 will help confirm the induction of tip colonies (Figure 2C) and CDPs (Figures 2D and 2F), respectively.

These iUB organoids are suitable for the generation of disease models for congenital anomalies of the kidney and urinary tract (CAKUT). For example, we developed a disease model for multicystic dysplastic kidney (MCDK) using our iUB organoids and heterozygous HNF1β-knockout hiPSC lines established by the CRISPR-Cas9 system (Bellanne-Chantelot et al., 2004; Heidet et al., 2010; Nakayama et al., 2010; Mae et al., 2020). These HNF1β^+/-^ iUB organoids exhibited fewer tip regions and faint apicobasal polarity, similar to the findings in MCDK model mice (Lokmane et al., 2010; Desgrange et al., 2017; Mae et al., 2020).

Thus, our iUB organoid system is valuable for generating *in vitro* CAKUT models and for elucidating the developmental mechanisms of UB branching and collecting duct maturation.

## Limitations

Our iUB organoids have some limitations. First, the absence of reciprocal interactions between hiPSC-derived nephron progenitors (NPs) and our iUB organoids prevents extensive branching morphogenesis of the iUB organoids and differentiation of the NPs into nephron structures in co-culture conditions (Mae et al., 2020). The absence of interactions may be caused by the functional immaturity of the hiPSC-derived NPs and iUB organoids or differences in the developmental stages between the NPs and iUB organoids.

Another limitation of our iUB organoid system is the difficulty in inducing mature collecting duct cells. Our CDPs are equivalent to their *in vivo* counterparts in human GW7-8 embryos (Wang et al., 2018). However, the branching morphogenesis of UB is repeated until GW15 or later in human embryonic kidneys (Costantini and Kopan, 2010; Potter, 1972; Osathanondh and Potter, 1963). Therefore, our iUB organoid system cannot be used to generate disease models for some types of CAKUT, especially cystic kidney diseases, because cystogenesis begins after collecting duct maturation.

## Troubleshooting

### Problem 1

Difficulty in inducing APS cells (step 3).

### Potential solution

**Figure 3:**
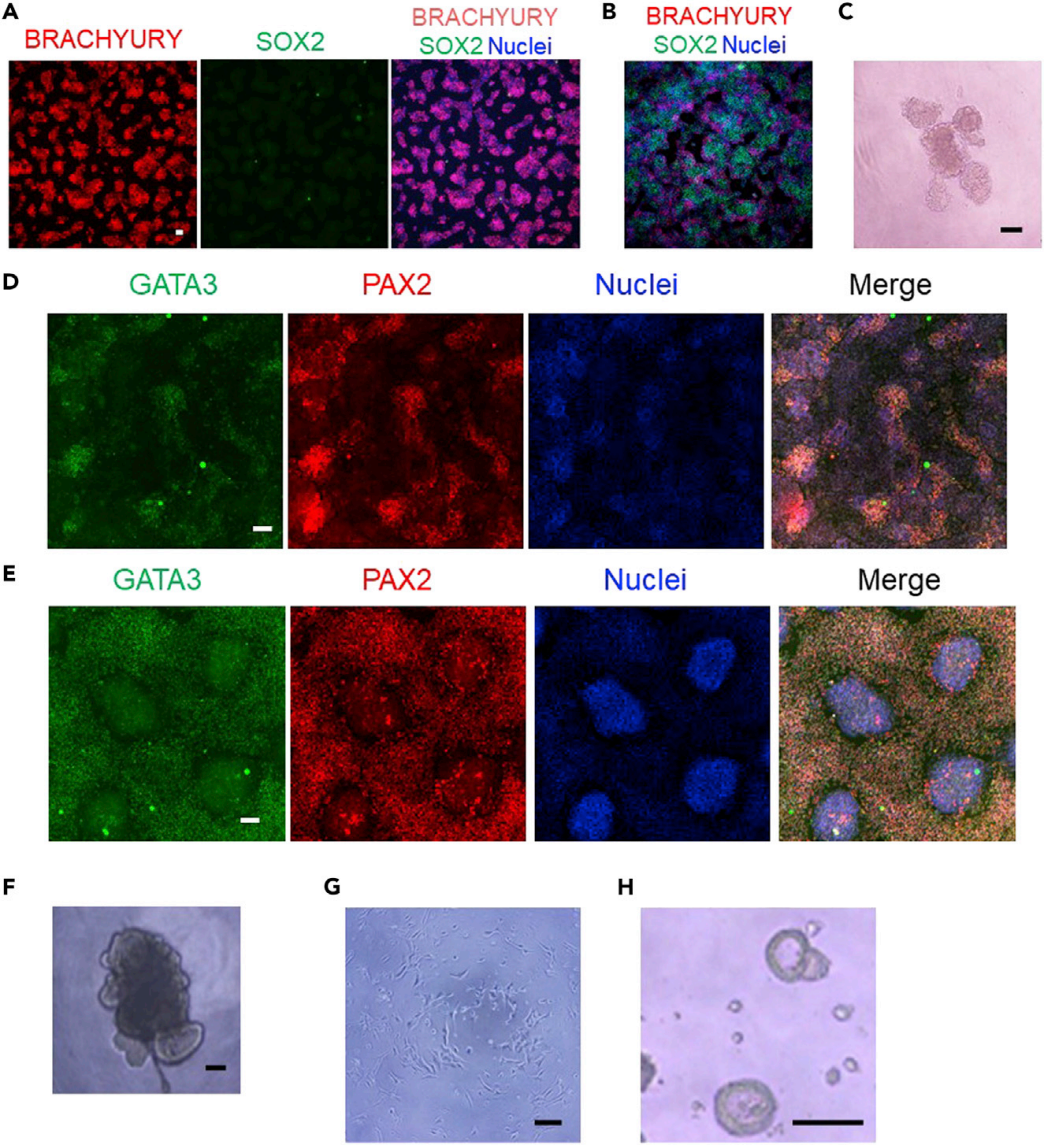
Troubleshooting (A and B) Immunostaining of day 0 (22 h) APS cells derived from two hiPSC lines, 1231A3 (A) and 1383D2 (B), for BRACHYURY (red), SOX2 (green) and nuclei (blue). Note that SOX2-positive hiPSCs remained in (B). (C) Morphology of a ND aggregate generated with low ND cell induction efficiency after manual pipetting. Note that the ND aggregate is destroyed. (D and E) Immunostaining of day 2 ND cells for GATA3 (green), PAX2 (red) and nuclei (blue). When the induction rate is low, GATA3- and PAX2-positive ND cells are distributed in an island shape (D). On the other hand, with a sufficient ND cell induction efficiency, GATA3- and PAX2-positive ND cells are more widespread and GATA3- and PAX2-negative cells are distributed in an island shape (E). (F) Morphology of a day 6 iUB organoid with reduced and thickened budding regions. (G) Morphology of incompletely formed tip colonies using too thin hydrogel or low concentrations of Matrigel. (H) Morphology of “halo”-like-shaped tip colonies formed with too long culture times or too many passages. Scale bars, 100 μm.

You may need to optimize the treatment time of Activin A and CHIR99021 because the time required for APS induction varies among hiPSC lines. Immunostaining analysis for BRACHYURY and SOX2 will help confirm the appropriate APS induction. APS cells will express BRACHYURY but not SOX2 (Figure 3A). The APS induction time is from 16 to 28 h. If the APS induction time is too short, SOX2-positive cells will remain in the center of the colonies (Figure 3B).

### Problem 2

When unwanted cells are separated from ND aggregates with manual pipetting, the aggregates break (step 7; Figure 3C).

### Potential solution

The induction rate of ND cells may be low (Figures 3D and 3E). You may reinduce ND cells from hiPSCs by improving the APS induction step (see troubleshooting 1).

### Problem 3

Budding regions of iUB organoids are reduced and/or thickened (step 7; Figure 3F).

### Potential solution

Matrigel concentrations may be too low to form the apicobasal polarity and tubular lumen of iUB organoids. You can increase the concentration of Matrigel up to 10%. vol in the **iUB organoid induction** step. You should gradually increase the concentration of Matrigel.

### Problem 4

No tip colonies are formed on the hydrogel (step 8; Figure 3G).

### Potential solution

The hydrogel layer may be too thin or the concentration of Matrigel may be too low to form tip colonies. Add up to 500 μL hydrogel to the 48-well plates or increase the concentrations of Matrigel up to 75% in the **UB tip colony induction** step.

### Problem 5

Failure to induce CDPs (step 9).

### Potential solution

When tip colonies look like halos (Figure 3H), their induction rate may be too low. Shorten the tip colony induction time to 5 days to increase the induction efficiency in the **UB tip colony induction** step.

## Resource availability

### Lead contact

Further information and requests for resources and reagents should be directed to and will be fulfilled by the lead contact, Kenji Osafune (osafu@cira.kyoto-u.ac.jp).

### Materials availability

This study did not generate new unique reagents.

## Data Availability

No new codes have been generated.

## References

[bib1] Bellanne-Chantelot C., Chauveau D., Gautier J.F., Dubois-Laforgue D., Clauin S., Beaufils S., Wilhelm J.M., Boitard C., Noel L.H., Velho G. (2004). Clinical spectrum associated with hepatocyte nuclear factor-1beta mutations. Ann. Intern. Med..

[bib2] Costantini F., Kopan R. (2010). Patterning a complex organ: branching morphogenesis and nephron segmentation in kidney development. Dev. Cell.

[bib3] Desgrange A., Heliot C., Skovorodkin I., Akram S.U., Heikkilä J., Ronkainen V.P., Miinalainen I., Vainio S.J., Cereghini S. (2017). HNF1B controls epithelial organization and cell polarity during ureteric bud branching and collecting duct morphogenesis. Development.

[bib4] Heidet L., Decramer S., Pawtowski A., Morinière V., Bandin F., Knebelmann B., Lebre A.S., Faguer S., Guigonis V., Antignac C., Salomon R. (2010). Spectrum of HNF1B mutations in a large cohort of patients who harbor renal diseases. Clin. J. Am. Soc. Nephrol..

[bib6] Lokmane L., Heliot C., Garcia-Villalba P., Fabre M., Cereghini S. (2010). vHNF1 functions in distinct regulatory circuits to control ureteric bud branching and early nephrogenesis. Development.

[bib7] Mae S.I., Ryosaka M., Sakamoto S., Matsuse K., Nozaki A., Igami M., Kabai R., Watanabe A., Osafune K. (2020). Expansion of human iPSC-derived ureteric bud organoids with repeated branching potential. Cell Rep..

[bib14] Mae S.I., Ryosaka M., Toyoda T., Matsuse K., Oshima Y., Tsujimoto H., Okumura s., Shibasaki A., Osafune K. (2018). Generation of branching ureteric bud tissues from human pluripotent stem cells. Biochem Biophys Res Commun.

[bib8] Miyazaki T., Isobe T., Nakatsuji N., Suemori H. (2017). Efficient adhesion culture of human pluripotent stem cells using laminin fragments in an uncoated manner. Sci. Rep..

[bib9] Nakayama M., Nozu K., Goto Y., Kamei K., Ito S., Sato H., Emi M., Nakanishi K., Tsuchiya S., Iijima K. (2010). HNF1B alterations associated with congenital anomalies of the kidney and urinary tract. Pediatr. Nephrol..

[bib10] Osathanondh V., Potter E.L. (1963). Development of human kidney as shown by microdissection. II. Renal pelvis, calyces, and papillae. Arch. Pathol..

[bib12] Potter E.L. (1972).

[bib13] Wang P., Chen Y., Yong J., Cui Y., Wang R., Wen L., Qiao J., Tang F. (2018). Dissecting the global dynamic molecular profiles of human fetal kidney development by single-cell RNA sequencing. Cell Rep..

